# Genetic Diversity and the Impact of the Breed Proportions of US Brown Swiss in German Brown Cattle

**DOI:** 10.3390/ani11010152

**Published:** 2021-01-11

**Authors:** Anna Wirth, Jürgen Duda, Ottmar Distl

**Affiliations:** 1 Institute of Animal Breeding and Genetics, University of Veterinary Medicine Hannover (Foundation), 30559 Hannover, Germany; anna.wirth@tiho-hannover.de; 2 Landeskuratorium der Erzeugerringe für Tierische Veredelung in Bayern e.V. (LKV), 80687 München, Germany; juergen.duda@lkv.bayern.de

**Keywords:** German Brown, inbreeding, ancestral inbreeding, probability of gene origin, genetic diversity, breed proportions of US Brown Swiss

## Abstract

**Simple Summary:**

The main aim of modern breeding programs in dairy cows is to improve productivity, functional and health traits. The use of only a few top sires leads to more efficient milk production, but also it could lead to a decline in the gene pool, smaller effective population size and an increase of inbreeding. Deleterious effects of inbreeding in dairy cattle may reduce the benefits of the genetic gains. Due to this fact, it is important to monitor the genetic diversity in dairy cattle breeds. In this study, pedigree data were used to show the losses of genetic variability and its association with the heavy use of imported US Brown Swiss bulls and semen in the German Brown population. Strategies to decrease rate of inbreeding through sires with less relationships to the most important ancestors should be considered in future breeding strategies.

**Abstract:**

Increase of inbreeding and loss of genetic diversity have large impact on farm animal genetic resources. Therefore, the aims of the present study were to analyse measures of genetic diversity as well as recent and ancestral inbreeding using pedigree data of the German Brown population, and to identify causes for loss of genetic diversity. The reference population included 922,333 German Brown animals born from 1990 to 2014. Pedigree depth and completeness reached an average number of complete equivalent generations of 6.24. Estimated effective population size for the German Brown reference population was about 112 with a declining trend from 141 to 95 for the birth years. Individual inbreeding coefficients increased from 0.013 to 0.036. Effective number of founders, ancestors and founder genomes of 63.6, 36.23 and 20.34 indicated unequal contributions to the reference population. Thirteen ancestors explained 50% of the genetic diversity. Higher breed proportions of US Brown Swiss were associated with higher levels of individual inbreeding. Ancestral inbreeding coefficients, which are indicative for exposure of ancestors to identical-by-descent alleles, increased with birth years but recent individual inbreeding was higher than ancestral inbreeding. Given the increase of inbreeding and decline of effective population size, measures to decrease rate of inbreeding and increase effective population size through employment of a larger number of sires are advisable.

## 1. Introduction

German Brown cattle with main breeding area in Southern Germany is a traditional dual-purpose breed with a strong focus on milk production and quality, longevity and feet and leg quality [1]. Historically, German Brown has its roots in grey-brown cattle populations including the Allgäuer and Württemberger Braunvieh, Graubraunes Tiroler Gebirgsrind [2] as well as Swiss and Austrian Brown cattle [3]. The introduction of US Brown Swiss bulls into the German Brown population started in 1966 and became part of the breeding strategy due to the enormous superiority in production efficiency of US Brown Swiss compared to German Brown cattle at the time [4]. As a result, the breed proportion of US Brown Swiss increased in today’s German Brown cows to about 70% [5]. In general, focusing on a small number of superior bulls leads unavoidably to an increase of inbred animals and thus to a decline of genetic variability in the population which can negatively influence the performance and reduce fitness traits in dairy cattle [6,7,8,9]. Thus, inbreeding coefficients based on pedigree data have been proven as a useful tool to evaluate the development of genetic diversity in a population. Previous studies on the level of inbreeding in German Brown cows showed an increase of the coefficient of inbreeding in the years 1980–1992 from 0.0032 to 0.0126 [10]. As with the classical approach the load of inbreeding in ancestors is not considered, ancestral inbreeding concepts have been developed some time ago [11,12,13], but rarely employed, e.g., in Irish and German Holsteins [9,14] as well as in German Red breeds [15]. A further approach to gain insight into the genetic variability is the use of the probabilities of gene origin that consider the genetic contributions of founders and ancestors [16]. This opens the possibility to detect bottlenecks and genetic drift in populations [16,17,18,19,20,21].

The objective of this study was to evaluate the genetic diversity of the German Brown population for the birth years from 1990 to 2014. Herein, we derived probabilities of gene origins and classical and ancestral coefficients of inbreeding from pedigree data. Due to the high breed proportions of US Brown Swiss in the German Brown population, we evaluated the influence of increasing breed proportions of US Brown Swiss on the degree of inbreeding.

## 2. Materials and Methods

The data for the present study were provided by the official milk recording organization of Bavaria (Landeskuratorium der Erzeugerringe für tierische Veredelung in Bayern e.V., LKV). Data included all available pedigree records of the German Brown population since electronic data recording started. The pedigree file employed for analysis contained 1,288,527 animals. For the analysis, only male and female animals with both known parents were included. Measures of genetic diversity and probabilities of gene origins were analysed in all first calving heifers born from 1990 to 2014 and all bulls mated with these cows. This data set was defined as reference population and included 908,228 cows and 14,105 bulls.

Data editing and calculation of effective population size, individual rate of inbreeding, coefficients of inbreeding in relation to the breed proportion of US Brown Swiss and broken line analysis for the trend of the breed proportion of US Brown Swiss with birth years were performed using SAS, version 9.4 (Statistical Analysis System, Cary, NC, USA, 2020). Pedigree analyses were carried out using the software package PEDIG [19].

The number of equivalent complete generations (GE), defined as the sum of the proportion of known ancestors over all generations was used to describe pedigree completeness. In addition, the percentages of known ancestors per generation were calculated [20].

Animals without known ancestors are defined as founders. Founders are expected to be unrelated with an inbreeding coefficient of zero. The effective number of founders, which is the number of equally contributing founders that would explain the genetic diversity of the reference population was calculated according to Lacy [21].

(1) $$f_{e}=1/\sum_{i=1}^{f}p_{i}^{2}$$

where *f_e_* is the effective number of founders, *f* the number of founders, and *p_i_* the expected genetic contribution of an individual founder *i* to the gene pool of the reference population. These probabilities over all founders sum to one. The *f_e_/f* ratio accounts for the degree of unequal founder contribution. Apart from the unlikely situation of equal founder contribution, the *f_e_/f* ratio is generally below 1. To account for bottleneck effects, the effective number of ancestors was calculated from the 1000 most contributing ancestors according to its marginal genetic contribution [16].

(2) $$f_{a}=1/\sum_{j=1}^{1000}q_{j}^{2}$$

where *f_a_* is the effective number of ancestors and *q_j_* is the marginal genetic contribution of ancestor *j* to the reference population. The marginal genetic contributions of ancestors are iteratively calculated whereby the first ancestor is chosen based on its expected genetic contribution, the following ancestors due to their marginal contributions, that is the contribution that has not been explained by an ancestor already considered in the calculation [16].

The ratio of *f_a_/f_e_* considers the population development since founder generation. Lower ratios reflect stronger effects of bottlenecks during population development, whereby the absence of bottlenecks may be assumed at values close to one.

The effective number of founder genomes was chosen to consider unequal contribution of founders as well as random losses of alleles. It is calculated equally to the effective number of founder by replacing a founder’s individual contribution by the contribution of founder genes [21].

(3) $$f_{g}=1/\sum_{i=1}^{f}\frac{p_{i}^{2}}{r_{i}}$$

where *f_g_* is the effective number of founder genomes, *f* is the number of founders, *p_i_* = proportion of the genes of founder *i*, which can be found in the reference population, *r_i_* = expected proportion of founder alleles that have been retained within the descendant population.

The ratio *f_g_/f_e_* shows the impact of drift with lower values indicating more loss of genetic diversity through drift.

To calculate the amount of genetic diversity (GD) accounting for loss of genetic diversity due to genetic drift and unequal contribution of founders we used following formula [22].

(4) $$\mathrm{GD}=1-\frac{1}{2f_{g}}$$

Correspondingly, the amount of genetic diversity (GD*) that accounts for loss of genetic diversity due to unequal contribution of founders was calculated as follows [22]

(5) $${\mathrm{GD}}^{*}=1-\;\frac{1}{2f_{e}}$$

The loss of genetic diversity due to genetic drift can be expressed as the difference between GD and GD*.

The inbreeding coefficient was calculated for the whole reference population and separately for inbred German Brown in the reference population using the algorithm developed by Meuwissen [23]. To analyse the development of the inbreeding coefficient in relation to the breed proportion of US Brown Swiss, the German Brown population was divided into 19 US Brown Swiss classes with intervals of 5%. This small subdivision was chosen as US Brown Swiss breed proportions are continuously increasing and a large number of animals have breed proportions of 60–80% US Brown Swiss.

In addition, the GRAIN package version 2.2 [13,24] was used to calculate individual inbreeding coefficients based on the gene dropping method (F_gd_) as well as ancestral inbreeding according to Ballou [11], Kalinowski [12] and an ancestral history coefficient (A_hc_) as defined by Baumung [13].

Ballou [11] defined the ancestral inbreeding coefficient (F_a_Bal_) as the cumulative proportion of an individual’s genome that has been previously exposed to inbreeding in its ancestors. According to this definition, inbreeding from each ancestor is taken into account and calculated independently of the individual inbreeding coefficient. Therefore, it is possible for an individual to have an individual inbreeding coefficient of zero and an ancestral inbreeding coefficient according to Ballou [11] greater than zero [11].

Kalinowski [12] divided the inbreeding coefficient in two parts. Ancestral inbreeding (F_a_Kal_), which means homozygous alleles have already met in former generations and new inbreeding (F_New_) where alleles are identical-by-descent (IBD) for the first time. As common ancestors have to be present on both sides of the pedigrees, F_a_Kal_ is zero if classical inbreeding is zero [12].

As Ballou’s concept of inbreeding [11] relies on all ancestors in the pedigree unlike its contribution to the individual inbreeding, F_a_Bal_ increases with every inbred ancestor in the pedigree. Thus, a steep rise of F_a_Bal_ compared to F_gd_ across years indicates an increase of inbred ancestors in the pedigrees, but these inbred ancestors do not contribute to the individual inbreeding coefficient. The ancestral history coefficient is a measure of how often a randomly taken allele has undergone IBD in the past during pedigree segregation [13].

Correlations between the different inbreeding coefficients and the breed proportions of US Brown Swiss were calculated using Pearson correlation coefficients with SAS, version 9.4 (Statistical Analysis System, Cary, NC, USA, 2020).

The individual rate of inbreeding per generation according to Gutièrrez [25] was considered to adjust for the pedigree depth.

(6) $$\Delta F_{i}=1-\sqrt[{GE_{i}\;-\;1}]{(1-F_{i}})$$

where ∆*F_i_* is the individual rate of inbreeding, *F_i_* is the inbreeding coefficient of individual *i*, *GE_i_* is the number of known equivalent generations for individual *i*.

The effective population size is the number of reproducing animals in an idealized population that would constitute the same genetic diversity as the population under study. It was calculated as realized effective population size [25] using the increase of individual inbreeding per generation [26].

(7) $$N_{e}=1/\left(2\Delta F_{i}\right)$$

where *N_e_* is the effective population size, and ∆*F_i_* is the individual rate of inbreeding per generation.

## 3. Results

For the birth years 1990–2014, the GE was 6.24 where a continuous increase over the birth years from 4.54 in 1990 to 8.04 in 2014 was observed. The mean proportion of known ancestors in generation 3, 5 and 7 was 94%, 82% and 46%, respectively (Supplementary Table S1). The results of the analysis of the probability of gene origins with *f_e_*, *f_a_* and *f_g_* are summarized in Table 1. The amount of genetic diversity lost in the reference population since the founder generation due to bottlenecks and genetic drift (1 − GD) was 0.025 and due to genetic drift 0.017 (GD* − GD). Loss through unequal contributions of founders (1 − GD*) reached 0.008. Relative contributions to loss of genetic diversity by drift and unequal use of founders were 68% and 32%, respectively.

The number of ancestors explaining 50%, 70% and 90% of the gene pool was 13, 36 and 751, respectively. The cumulated marginal contribution of the 10 most contributing ancestors amounted to 0.44 (Table 2).

The average inbreeding coefficient for all and only inbred German Brown was 0.023 and 0.026, respectively. The different average ancestral inbreeding coefficients ranged from 0.002 to 0.023 with F_a_Kal_ at 0.002 and A_hc_ at 0.023 (Table 3).

**Figure 1:**
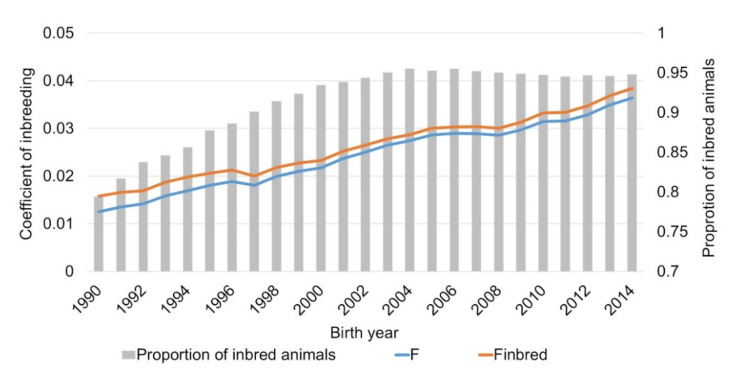
Inbreeding coefficients for all (F) and inbred (F_inbred_) German Brown for the birth years 1990 to 2014.

The proportion of inbred animals in the reference population increased from the birth year 1990 to 2002 nearly linear from 79% to 95% and remained roughly at 95% for the period from 2003 to 2014. For the average coefficient of inbreeding an increase over time from 0.013 and 0.016 in 1990 to 0.036 and 0.038 in 2014 was observed for all German Brown and inbred German Brown, respectively (Figure 1).

**Figure 2:**
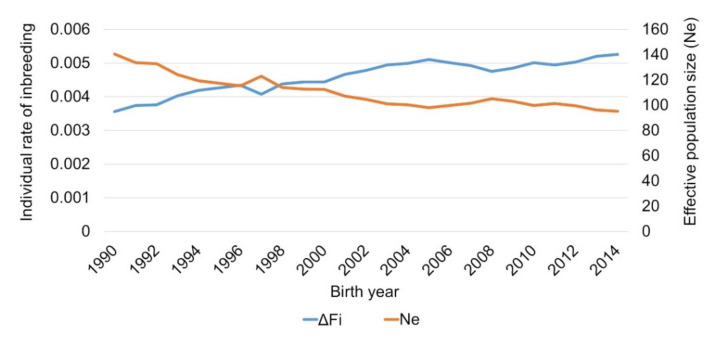
Effective population size (N_e_) and individual rate of inbreeding per generation (ΔF_i_) for German Brown born between 1990 and 2014.

The same trend was seen for the individual rate of inbreeding per generation with an increase from 0.0036 to 0.0053 from 1990 to 2014. A slight decrease in the individual rate of inbreeding per generation was obvious in 1997, 2006–2008 and in 2011. The effective population size decreased inversely to the individual rate of inbreeding per generation from 141 in 1990 to 95 in 2014 (Figure 2).

**Figure 3:**
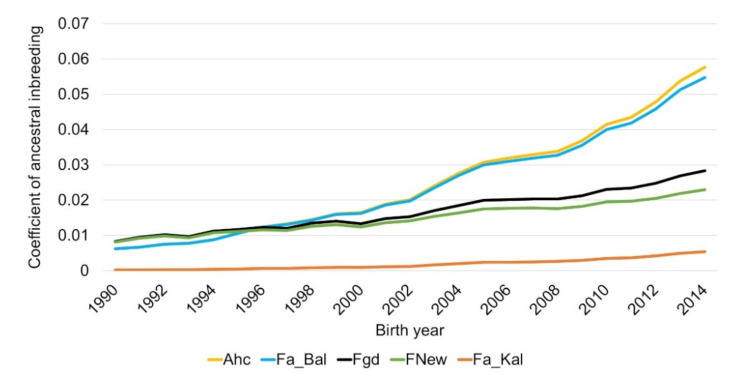
Ancestral inbreeding according to Ballou (F_a_Bal_), ancestral (F_a_Kal_) and new (F_New_) inbreeding according to Kalinowski and ancestral history coefficient according to Baumung (A_hc_) as well as individual inbreeding coefficient based on the gene dropping method (F_gd_) for German Brown for the birth years 1990 to 2014.

All ancestral coefficients of inbreeding showed an increasing trend over time. The highest increase was determined for A_hc_ and F_a_Bal_, which reached nearly 0.06 in 2014, whereas F_gd_ reached 0.028 in 2014. Compared to F_gd_, a lower increase was found in F_New_ and F_a_Kal_, whereas the level of F_New_ was higher compared to F_a_Kal_ (Figure 3).

The correlations between the different inbreeding coefficients are presented in Table 4. We observed high correlations between F_a_Bal_ and A_hc_ and between F_New_ and F as well as F_gd_, whereas correlations were low between F_New_ and F_a_Bal_ as well as A_hc_.

**Figure 4:**
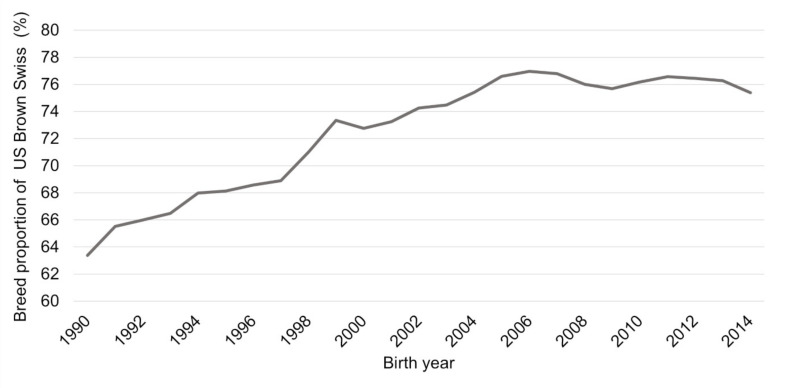
Breed proportions of US Brown Swiss for German Brown born between 1990 and 2014.

The breed proportions of US Brown Swiss raised from 63% in 1990 to 77% in 2006, whereas from 2007 on, a slight declining tendency to 2014 to 75% was seen (Figure 4). Using a broken line model with the procedure NLIN of SAS, version 9.4 (Statistical Analysis System, Cary, NC, USA, 2020), the yearly increase was 0.8393 to 76.71% of breed proportions of US Brown Swiss in 2006 and from there a yearly decrease by −0.0923 to 75.9% breed proportions of US Brown Swiss.

**Figure 5:**
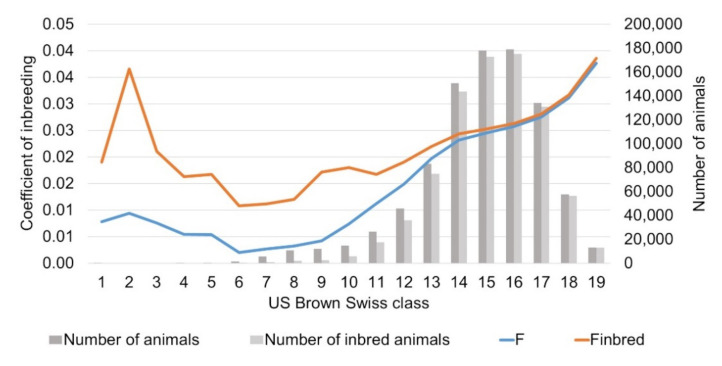
Number of German Brown and inbred German Brown and coefficient of inbreeding for all (F) and inbred (F_inbred_) German Brown by breed proportions of US Brown Swiss in classes of 5%.

About three quarters of the reference population had a breed proportion of US Brown Swiss > 70%. The analysis of inbreeding in relation to the breed proportion of US Brown Swiss revealed an increasing coefficient of inbreeding with increasing breed proportion of US Brown Swiss. Animals having a breed proportion of US Brown Swiss >90% showed a mean coefficient of inbreeding >0.03. They represent 8% of the German Brown animals in the reference population (Figure 5).

## 4. Discussion

For the German Brown population, the development of genetic diversity including birth years from 1990 to 2014 different measures of inbreeding were analysed. Pedigree depth was assessed using the GE and the proportion of known ancestors per generation. The average GE in the current study was 6.24 with an increasing trend from 1990 to 2014 for the reference population. Therefore, we used the individual rate of inbreeding per generation for calculation of the effective population size. For Swiss Brown in Switzerland, GE increased from 6.9 in 1992 to 8.1 in 2002 [17]. Sørenson et al. [27] reported GE for Danish Holstein, Jersey and Danish red ranging from 6.7 to 7.36. Studies using a smaller number of animals revealed 5.8 GE for French Brown Swiss from 2004 to 2007 [18], 8.7 for Canadian Brown Swiss [22] and 4.82 for German Holstein [28]. The percentage of known ancestors per generations were higher than those found for Canadian Brown Swiss [22] and German Red Angler [15]. Regarding analysis of genetic diversity based on pedigree information, pedigree quality is crucial to avoid underestimation of inbreeding measures [16,29].

The N_e_ was calculated with the individual rate of inbreeding per generation and is thus less susceptible to overestimation due to low pedigree depth [25]. In French Brown Swiss N_e_ was slightly smaller with 98 [18], in Canadian Brown Swiss much smaller with estimates at 47–76 [22] as well as in Brown cattle from Switzerland with 70.6 [17]. A study for the international Brown Swiss populations with 71,497 bulls from 22 countries gave a mean coefficient of inbreeding of 0.0077 and mean N_e_ between 63 and <150 for the birth years 1995–2003 and 204 for the birth year 2004 [30]. In monitoring programs for conserving genetic diversity, N_e_ is the most important criterion for evaluation the endangerment status of a population. Effective size should be larger than 50–100 to maintain the critical potential to withstand adverse effects due to inbreeding [31]. In the long-term, a N_e_ of 500 was proposed for a sustainable development of a population [32]. The decrease in N_e_ from 1990 to 2014 to a N_e_ of 95 is the result of an increasing individual rate of inbreeding per generation. This indicates the need of a long-term surveillance of genetic variability in order to decrease the rate of inbreeding in German Brown and prevent further loss of genetic diversity. A more balanced use of breeding bulls and highlighting coancestries among breeding bulls and mating partners as well as expected future inbreeding in progeny relative to the active population may help to constrain the rate of inbreeding at a lower level.

Useful parameters to describe the genetic variability of a population are those derived from the probabilities of gene origin. The *f_e_* differed clearly from the number of founders as equal contributions are unlikely in livestock populations. In French Brown Swiss, *f_e_* was 79 and between 65 and 81 for French Holstein, Montbéliarde and Normande for the birth years 2004 to 2007 [18]. Comparable estimates were found in Danish Holstein with 70 effective founders [27] and Canadian Jersey with 68 and 70 effective founders for cows born between 2000–2006 and bulls from 1998–2000, respectively [33]. Somewhat higher estimates were reported for Canadian Brown Swiss (120) [22], German Holstein (93–111) [28], Irish Holstein (112) [34] and Tunisian Holstein (112–194) [35]. The *f_a_*/*f_e_* ratio was 0.57, indicating the presence of recent bottlenecks in the German Brown population and random loss of genetic diversity due to a declining number of effectively contributing animals [27]. Considerably lower ratios were reported for French Brown Swiss cows born between 2004 and 2007, active males and cryobank males with 0.35, 0.26 and 0.33, respectively [18]. The ratio of *f_a_*/*f_e_* in Canadian Brown Swiss born between 2003–2007 was 0.24 [22], 0.21 for two German Holstein reference populations born between 1998–2002 and 2003–2007 [28] and 0.29–0.36 for German Red breeds [15] (Table S2). Reasons for this higher ratio of *f_a_*/*f_e_* in the current German Brown population may be due to the steep decrease of *f_e_* as a consequence of the immigration of US Brown Swiss bulls starting in 1966 and continuing to the present. The *f_e_/f* ratio indicated a strong unbalanced contribution of founders caused by the nearly exclusive use of a low number US Brown Swiss bulls and bulls with high percentage of US Brown Swiss blood. Thus, the paternal contribution to the genetic diversity of the original German Brown eroded and was largely replaced by a smaller proportion of US Brown Swiss genetics [4]. Crossbreeding with US Brown Swiss bulls should have increased genetic diversity in the case a larger number of immigrant bulls would have been employed and thus, coancestry among bulls in later generations would have been kept at a lower level.

An *f_g_/f_e_* ratio of 0.32 implies loss of genetic diversity due to random genetic drift independently from founder contributions. In Canadian Brown Swiss, German and Canadian Holstein an even smaller ratio indicative for more impact of genetic drift was obtained [22,28,33] (Supplementary Table S2). In addition, the relative contribution of genetic drift on the loss of genetic diversity in the German Brown population was 68%, whereas contrasting results were shown for Canadian Brown Swiss with nearly 90% [22] and 95% in Canadian Holstein [33]. This lower impact of random genetic drift on the loss of genetic diversity in the German Brown population may be the result of a less unequal diffusion of founder genomes in the German Brown population. Genetic drift naturally occurs in each generation and it increases through strict selection strategies and mating of more closely related animals. In the German Brown population, both, purebred US Brown Swiss bulls and crossbreds with German Brown cows were very popular and important founders in the reference population. This means genetic diversity caused by the large number of original German Brown bulls before incrossing began was lost in the first crossbred generations, but on the other hand loss of founder genomes was counterbalanced through an increasing number of bulls with varying breed proportions of US Brown Swiss. Therefore, loss due to drift was reduced.

Increasing of inbreeding inevitably occurs in dairy cattle with commercial breeding programs. This was shown for the German Brown population with an increasing coefficient of inbreeding as well as an increasing individual rate of inbreeding. The level of inbreeding observed in this study is comparable with the development of inbreeding found in European Brown Swiss bulls from 1990 to 2004 from 0.0165 to 0.035 [30] and the nearly linear increase in average inbreeding found in Swiss Brown from 1992 to 2002 from nearly 0.02 to 0.035 [17]. Furthermore, the average inbreeding of 0.026 found in French Brown from 2004–2007 is in the range of the results from the German Brown, indicating similar development of the genetic diversity of the European Brown breeds [18]. Lower levels of inbreeding had been reported in German Brown from 1980 to 1992 [10] and Swiss Brown from 1960 to 1984 [36]. Pedigree data of three large German Holstein herds revealed higher inbreeding coefficients with estimates at 0.0325 for inbred animals [14]. In Czech Holsteins, inbreeding coefficients increased from 1996 to 2013 from 0.013 to 0.05 [37]. For Dutch Holsteins, mean inbreeding coefficient was 0.05 based on pedigrees with a mean GE of 12.5 [38]. North American Holsteins and Jerseys from birth years of 1990 to 2018 with pedigree completeness indices at 98.8 to 99.9% showed inbreeding coefficients of 0.0774 and 0.072, respectively [39]. Much lower inbreeding coefficients were reported for German Red breeds ranging from 0.0075 to 0.0139 [16]. Inbreeding coefficients of 13,712 Finnish Red animals born between 2002 and 2014 were at 0.02–0.03 [40].

Only a few studies have dealt with ancestral inbreeding in dairy cattle populations yet [9,14,15]. Ballou’s ancestral coefficient of inbreeding [11] in this study was lower compared to the results for the German and Irish Holstein and German Angler respectively [9,14,15]. Lower values compared to the present study were found in the German Red and White Dual purpose breed [15]. A higher increase of F_a_Bal_ compared to the inbreeding derived from the gene drop method was also seen in German Holsteins [14]. The mean A_hc_ [13] and its trend over the birth years equals F_a_Bal_ which is in agreement with the findings in the German Red breeds [15]. The high correlation between F_a_Bal_ and A_hc_ is indicating similar basic assumptions of these approaches. Kalinowski [12] distinguished the ancestral (F_a_Kal_) and the new part (F_New_) of inbreeding. F_a_Kal_ can only be calculated when common ancestors are found on both sides of the pedigree. F_a_Kal_ and F_New_ take smaller values compared to the individual inbreeding coefficient and F_gd_. The reason for this stems from the definition of F_a_Kal_ and F_New_, which are defined in such a way that they sum up to the individual inbreeding coefficient. As F_New_ was higher in the present study than F_a_Kal_, most inbreeding may have occurred recently. In German Holsteins, F_New_ and F_a_Kal_ were at a higher level with 0.0256 and 0.0068, respectively [14]. However, in Irish Holsteins, inbreeding occurred in earlier generations as F_a_Kal_ was much higher than F_New_ [9]. Regarding the proportion of F_New_ and F_a_Kal_, the German Brown population is more severely influenced by new inbreeding than German Holsteins from three large dairy farms. In Dutch Holsteins F_a_Kal_ was only slightly higher than F_New_, indicating inbreeding has occurred anciently and recently to a similar degree [38]. Hinrichs et al. [14] suggested differences in pedigree structure and depth as well as different breeding goals may have an effect to such outcomes. Especially in the Dutch study, a GE of 12.5 represents deeper pedigrees compared to all other studies and this may explain some differences in the outcomes [38]. In general, new inbreeding is thought to be more detrimental compared to ancient inbreeding, as an allele that has occurred in an individual’s pedigree many times throughout the pedigree is expected to have a neutral or even positive effect compared to those being IBD more recently [38]. The correlation between F and F_a_Kal_ was higher than that between F and F_a_Bal_. Similar results were found in German and Irish Holsteins as well as German Red breeds, but correlations were higher and varied from 0.75 to 0.99 and 0.36 to 0.77, respectively [9,14,15]. Correlation between F_a_Bal_ and F_a_Kal_ in this study was high and in the range of other studies suggesting a relationship between those parameters [14,15]. The high correlations between F and F_gd_ with F_New_ are in agreement with the studies on Irish and German Holstein [9,14]. Differences in correlations between the populations may be a result of different population structures. The correlations between the different coefficients of inbreeding and the breed proportions of US Brown Swiss were moderate. Ancestral inbreeding coefficients showed higher correlations with the breed proportions of US Brown Swiss than F_New_ and individual inbreeding coefficients indicating steeper increase of inbreeding through immigrating US Brown Swiss bulls in ancestral generations. A high breed proportion of US Brown Swiss was accompanied by an increasing rate of inbreeding per generation. The higher level of inbreeding in the lower US Brown Swiss classes 1–5, representing a breed proportion of US Brown Swiss up to 30%, may be a result of the low number of animals in these classes or selective breeding for cows with lower breed proportion of US Brown Swiss. Crossings with bulls from another population should increase genetic diversity. Reasons for the contrary results may be the continued overproportional use of a low number of US Brown Swiss bulls and bulls with high breed proportions of US Brown Swiss. Particularly, when these bulls show higher degrees of coancestries as well as advantages in selection of cows with higher breed proportions of US Brown Swiss, coefficients of inbreeding increase along with breed proportions of US Brown Swiss.

## 5. Conclusions

Results of this study indicate a loss of genetic diversity in the German Brown population due to drift and unequal founder contributions. In comparison to other Brown Swiss and Holstein populations, losses due to genetic drift were less important. Balanced contributions of sires in the current breeding program may therefore maintain genetic diversity and prevent a further decline of N_e_ in the German Brown population. Differentiating individual inbreeding coefficients into their ancestral and new parts according to Kalinowski [12], most part of inbreeding was attributable to recent inbreeding. The correlation between increasing breed proportions of US Brown Swiss and higher levels of inbreeding indicates an influence of the selection program on genetic diversity. Therefore, given the increasing individual rate of inbreeding per generation through gene flow from US Brown Swiss bulls, measures that maintain the genetic diversity in German Brown should be implemented through the breeding program.

## Figures and Tables

**Table 1:** Results of the pedigree analysis for measures of genetic diversity for the German Brown population of the birth years from 1990 to 2014.

Parameter	Number
Reference population	922,333
Inbred animals in the reference population	839,058
Equivalent complete generations (GE)	6.24
Number of founders (*f*)	96,696
Effective number of founders (*f_e_*)	63.6
Effective number of ancestors (*f_a_*)	36.23
Effective number of founder genomes (*f_g_*)	20.34
*f_a_/f_e_*	0.57
*f_g_/f_e_*	0.32
Ancestors explaining 30% of the gene pool	4
Ancestors explaining 40% of the gene pool	8
Ancestors explaining 50% of the gene pool	13
Ancestors explaining 60% of the gene pool	20
Ancestors explaining 70% of the gene pool	36
Ancestors explaining 80% of the gene pool	82
Ancestors explaining 90% of the gene pool	751
Effective population size (N_e_)	111.7

**Table 2:** Ancestors with the greatest marginal contributions on the reference population.

ID	Birth Date	Name	Breed Proportion of US-BS (%)	Marginal Contribution	Cumulated Marginal Contributions	Number of Progeny
103,245	11.01.1966	ELEGANT	100	0.0902	0.0902	181
97,456	04.02.1956	LADDIE	100	0.0710	0.1612	43
128,376	02.05.1972	STRETCHER	100	0.0563	0.2175	881
284,689	16.03.1987	VINOS	67	0.0513	0.2688	18,803
105,963	18.08.1967	NORVICUS	100	0.0395	0.3083	3804
153,146	28.04.1976	ZELAD	88	0.0320	0.3403	7210
97,530	14.10.1960	NORVIC	100	0.0296	0.3699	108
100,987	02.06.1965	BRITE	100	0.0262	0.3961	264
126,464	31.07.1972	LADKUS	63	0.0209	0.4170	5540
248,754	11.10.1984	EMORY	100	0.0208	0.4378	1445

All ancestors are male.

**Table 3:** Average inbreeding coefficient for all animals (F) and inbred animals (F_inbred_), individual rate of inbreeding per generation (ΔF_i_), individual inbreeding using the genedrop method (F_gd_) and ancestral inbreeding according to Ballou (F_a_Bal_) and Kalinowski (F_a_Kal_), new inbreeding according to Kalinowski (F_New_) and ancestral history coefficient according to Baumung (A_hc_) for German Brown from the birth years 1990 to 2014.

Parameter	Inbreeding Coefficients
F	0.023
F_inbred_	0.026
ΔF_i_	0.005
F_gd_	0.016
F_a_Bal_	0.022
F_a_Kal_	0.002
F_New_	0.014
A_hc_	0.023

**Table 4:** Correlations between inbreeding coefficients according to Meuwissen (F), individual inbreeding using the gene drop method (F_gd_), ancestral inbreeding according to Ballou (F_a_Bal_) and Kalinowski (F_a_Kal_), new inbreeding according to Kalinowski (F_New_) and ancestral history coefficient according to Baumung (A_hc_) and the breed proportions of US Brown Swiss (BS) for German Brown born between 1990 and 2014. All correlation coefficients showed *p*-values < 0.0001.

	F	F_gd_	F_a_Bal_	A_hc_	F_a_Kal_	F_New_	BS
F		0.94	0.38	0.38	0.63	0.93	0.37
F_gd_			0.36	0.36	0.65	0.99	0.32
F_a_Bal_				1.00	0.72	0.27	0.44
A_hc_					0.72	0.27	0.44
F_a_Kal_						0.55	0.38
F_New_							0.29

## Data Availability

Restrictions apply to the availability of these data. Data were obtained from the Landeskuratorium der Erzeugerringe für tierische Veredelung in Bayern e.V. and are available from the authors with the permission of the Landeskuratorium der Erzeugerringe für tierische Veredelung in Bayern e.V.

## Supplementary Materials

The following are available online at https://www.mdpi.com/2076-2615/11/1/152/s1: Table S1. Number of complete equivalent generations (GE), number of animals and proportion of known ancestors for generations 1 to 10 for German Brown born from 1990 to 2014. Table S2. Results from the analysis of probability of gene origins from previous reports including effective population size (N_e_), number of founders (*f*), effective number of founders (*f_e_*), effective number of ancestors (*f_a_*), effective number of founder genomes (*f_g_*) and their corresponding ratios.
