# Supplementary material for: Genetic Diversity and the Impact of the Breed Proportions of US Brown Swiss in German Brown Cattle
Source: Animals (Basel). 2021 Jan 11;11(1):152. doi: 10.3390/ani11010152 (PMC7828010; doi:10.3390/ani11010152)
Supplement: Supplementary file 1 [file animals-11-00152-s001.zip › Table S1.docx]

**Table S1.** Number of complete equivalent generations (GE), number of animals and proportion of known ancestors for generations 1 to 10 for German Brown born from 1990 to 2014

| Birth year | GE | Number | Generation | | | | | | | | | |
| --- | --- | --- | --- | --- | --- | --- | --- | --- | --- | --- | --- | --- |
|  |  |  | 1 | 2 | 3 | 4 | 5 | 6 | 7 | 8 | 9 | 10 |
| 1990 | 4.54 | 41404 | 1 | 0,94 | 0,89 | 0,79 | 0,55 | 0,27 | 0,08 | 0,01 | 0,00 | 0,00 |
| 1991 | 4.64 | 41587 | 1 | 0,94 | 0,89 | 0,80 | 0,58 | 0,30 | 0,10 | 0,02 | 0,00 | 0,00 |
| 1992 | 4.79 | 41392 | 1 | 0,94 | 0,89 | 0,83 | 0,62 | 0,34 | 0,13 | 0,03 | 0,01 | 0,00 |
| 1993 | 4.96 | 40458 | 1 | 0,94 | 0,90 | 0,85 | 0,66 | 0,39 | 0,17 | 0,05 | 0,01 | 0,00 |
| 1994 | 5.08 | 44303 | 1 | 0,94 | 0,90 | 0,87 | 0,69 | 0,43 | 0,19 | 0,06 | 0,01 | 0,00 |
| 1995 | 5.27 | 46275 | 1 | 0,94 | 0,91 | 0,88 | 0,74 | 0,48 | 0,23 | 0,07 | 0,02 | 0,00 |
| 1996 | 5.38 | 44475 | 1 | 0,95 | 0,91 | 0,88 | 0,76 | 0,51 | 0,26 | 0,09 | 0,02 | 0,00 |
| 1997 | 5.47 | 42521 | 1 | 0,95 | 0,90 | 0,87 | 0,77 | 0,54 | 0,29 | 0,11 | 0,03 | 0,01 |
| 1998 | 5.59 | 41880 | 1 | 0,96 | 0,91 | 0,88 | 0,78 | 0,56 | 0,31 | 0,13 | 0,04 | 0,01 |
| 1999 | 5.78 | 39570 | 1 | 0,96 | 0,93 | 0,90 | 0,82 | 0,61 | 0,35 | 0,15 | 0,05 | 0,01 |
| 2000 | 5.95 | 39593 | 1 | 0,97 | 0,93 | 0,90 | 0,85 | 0,65 | 0,38 | 0,18 | 0,06 | 0,01 |
| 2001 | 6.13 | 38940 | 1 | 0,97 | 0,94 | 0,91 | 0,87 | 0,69 | 0,44 | 0,21 | 0,08 | 0,02 |
| 2002 | 6.29 | 35301 | 1 | 0,97 | 0,95 | 0,92 | 0,88 | 0,73 | 0,48 | 0,24 | 0,09 | 0,02 |
| 2003 | 6.42 | 35146 | 1 | 0,98 | 0,96 | 0,92 | 0,89 | 0,75 | 0,51 | 0,27 | 0,11 | 0,03 |
| 2004 | 6.57 | 35929 | 1 | 0,98 | 0,96 | 0,93 | 0,90 | 0,78 | 0,54 | 0,30 | 0,13 | 0,04 |
| 2005 | 6.68 | 34375 | 1 | 0,98 | 0,96 | 0,94 | 0,90 | 0,80 | 0,57 | 0,33 | 0,15 | 0,05 |
| 2006 | 6.86 | 34124 | 1 | 0,98 | 0,96 | 0,94 | 0,91 | 0,82 | 0,62 | 0,37 | 0,18 | 0,06 |
| 2007 | 6.95 | 32705 | 1 | 0,98 | 0,96 | 0,93 | 0,90 | 0,82 | 0,65 | 0,40 | 0,20 | 0,08 |
| 2008 | 7.08 | 32992 | 1 | 0,98 | 0,96 | 0,92 | 0,89 | 0,83 | 0,68 | 0,45 | 0,24 | 0,10 |
| 2009 | 7.21 | 32646 | 1 | 0,98 | 0,96 | 0,92 | 0,89 | 0,84 | 0,71 | 0,48 | 0,26 | 0,11 |
| 2010 | 7.37 | 31112 | 1 | 0,97 | 0,96 | 0,93 | 0,90 | 0,85 | 0,74 | 0,52 | 0,30 | 0,13 |
| 2011 | 7.48 | 30523 | 1 | 0,97 | 0,96 | 0,94 | 0,90 | 0,86 | 0,75 | 0,55 | 0,32 | 0,15 |
| 2012 | 7.63 | 29717 | 1 | 0,97 | 0,96 | 0,94 | 0,90 | 0,87 | 0,77 | 0,59 | 0,36 | 0,17 |
| 2013 | 7.82 | 27954 | 1 | 0,97 | 0,96 | 0,95 | 0,91 | 0,87 | 0,80 | 0,63 | 0,41 | 0,21 |
| 2014 | 8.04 | 27411 | 1 | 0,97 | 0,96 | 0,95 | 0,92 | 0,88 | 0,82 | 0,68 | 0,46 | 0,25 |
