# Supplementary material for: Genetic Diversity and the Impact of the Breed Proportions of US Brown Swiss in German Brown Cattle
Source: Animals (Basel). 2021 Jan 11;11(1):152. doi: 10.3390/ani11010152 (PMC7828010; doi:10.3390/ani11010152)
Supplement: Supplementary file 1 [file animals-11-00152-s001.zip › Table S2 rev.docx]

**Table S2**. Results from the analysis of probability of gene origin from the literature with the effective population size (N_e_) the number of founders (f), the effective number of founders (f_e_), the effective number of ancestors (f_a_),the effective number of founder genomes (f_g_) and the corresponding ratios of the literature

| **Reference** | **Country** | **breed** | **Birth year** | **N (ref.pop)** | **N_e_** | ***f*** | ***f_e_*** | ***f_a_*** | ***f_g_*** | ***f_a_/f_e_*** | ***f_g_/f_e_*** |
| --- | --- | --- | --- | --- | --- | --- | --- | --- | --- | --- | --- |
| Addo et al. (2017) [15] | GER | RVA |  | 76,520 | 156 |  | 310 | 90 |  | 0.29 |  |
| Addo et al. (2017) [15] | GER | RND |  | 73,749 | 170 |  | 519 | 189 |  | 0.36 |  |
| Danchin-Burge et al. (2012) [18] | FRA | HOL | 2004-2000^C^ | 2,099,307 | 48-93 | 662,093 | 82 | 21 |  | 0.26 |  |
| Danchin-Burge et al. (2012) [18] | FRA | HOL | ^B^ | 868 |  |  | 67 | 14 |  | 0.21 |  |
| Danchin-Burge et al. (2012) [18] | FRA | MO | 2004-2007^C^ | 450,214 | 51-82 | 132,874 | 65 | 19 |  | 0.29 |  |
| Danchin-Burge et al. (2012) [18] | FRA | MO | ^B^ | 528 |  |  | 57 | 14 |  | 0.25 |  |
| Danchin-Burge et al. (2012) [18] | FRA | NO | 2004-2007^C^ | 329,272 | 55-92 | 106,206 | 81 | 23 |  | 0.28 |  |
| Danchin-Burge et al. (2012) [18] | FRA | NO | ^B^ | 398 |  |  | 69 | 16 |  | 0.23 |  |
| Danchin-Burge et al. (2012) [18] | FRA | BS | 2004-2007^C^ | 19,266 | 70-98 | 7292 | 79 | 28 |  | 0.35 |  |
| Danchin-Burge et al. (2012) [18] | FRA | BS | ^B^ | 132 |  |  | 43 | 11 |  | 0.26 |  |
| Hammami et al. (2007) [354] | TUN | HOL | 1990-2000 | 47,321 |  | 10,798^2^ | 112^2^ | 22^2^ |  | 0.20^1,2^ |  |
| Hammami et al. (2007) [354] | LUX | HOL | 1990-2000 | 75,467 |  | 9,328^2^ | 295^2^ | 44^2^ |  | 0.15^1,2^ |  |
| Hinrichs und Thaller (2011) [28] | GER | HOL | 1998-2002 | 19,537 |  |  | 111.3 | 23.4 | 14.10 | 0.21^1^ | 0.13^1^ |
| Hinrichs und Thaller (2011) [28] | GER | HOL | 2003-2007 | 19,060 |  |  | 92.8 | 19.23 | 11.01 | 0.21^1^ | 0.12^1^ |
| Mc Parland et al. (2007) [344] | IRE | HOL | 2004 | 233,986 | 75 | 60,474 | 112 | 40 | 24 | 0.36^1^ | 0.21^1^ |
| Melka et al. (2013) [22] | CAN | A | 2003-2007 | 49,354 | 54 | 6,307 | 40 | 13 | 7 | 0.32 | 0.17 |
| Melka et al. (2013) [22] | CAN | BS | 2003-2007 | 7,630 | 47 | 2,335 | 120 | 29 | 9 | 0.24 | 0.07 |
| Melka et al. (2013) [22] | CAN | CN | 2003-2007 | 1,099 | 40 | 293 | 59 | 10 | 5 | 0.17 | 0.08 |
| Melka et al. (2013) [22] | CAN | GUE | 2003-2007 | 1,557 | 46 | 2,018 | 87 | 13 | 8 | 0.15 | 0.09 |
| Melka et al. (2013) [22] | CAN | MS | 2003-2007 | 1,165 | 66 | 975 | 206 | 27 | 17 | 0.13 | 0.08 |
| Sørensen et al. (2005) [27] | DNK | HOL | 1960-2003 | 1,883,983 | 49 |  | 70 | 20.6 | 11.9 | 0.29^1^ | 0.17^1^ |
| Sørensen et al. (2005) [27] | DNK | J | 1960-2003 | 336,966 | 53 |  | 115.7 | 23.8 | 11.4 | 0.21^1^ | 0.10^1^ |
| Sørensen et al. (2005) [27] | DNK | DR | 1960-2003 | 261,047 | 47 |  | 207.2 | 34.6 | 21.7 | 0.17^1^ | 0.10^1^ |
| Stachowicz et al. (2011) [33] | CAN | HOL | 2000-2007^C^ |  | 114 |  | 293 | 16 | 7.7 | 0.05^1^ | 0.03^1^ |
| Stachowicz et al. (2011) [33] | CAN | HOL | 1998-2006^B^ |  |  |  | 264 | 14 | 5.9 | 0.05 | 0.02 |
| Stachowicz et al. (2011) [33] | CAN | J | 2000-2006^C^ |  | 54 |  | 67.8 | 18.9 | 8.2 | 0.28^1^ | 0.12^1^ |
| Stachowicz et al. (2011) [33] | CAN | J | 1998-2000 ^B^ |  |  |  | 69.9 | 19.5 | 6.1 | 0.28^1^ | 0.09^1^ |
| Worede et al. (2013) [30] | CH | BS | 1950-2004 | 138,165 | 63-204 | 41,011 | 141 | 88 |  | 0.61^1^ |  |

^1^calculated from original values in the reference, ^2^referes to birth year 2000, ^B^ only bulls, ^C^ only cows, A = Ayrshire, BS = Brown Swiss, CN = Canadienne, DR = Danish Red, GUE = Guernsey, HOL = Holstein, J = Jersey, MO = Montbéliarde, MS = Milking Shorthorn, NO = Normande, RVA = Red Angler, RND = Red-and-White Dual-purpose; CAN = Canada, CH = Switzerland, DNK = Denmark, FRA = France, GER = Germany, IRE = Ireland LUX = Luxembourg, TUN = Tunisia
